# Proteomic Analysis of the Effect of Korean Red Ginseng in the Striatum of a Parkinson’s Disease Mouse Model

**DOI:** 10.1371/journal.pone.0164906

**Published:** 2016-10-27

**Authors:** Dongsoo Kim, Hyongjun Jeon, Sun Ryu, Sungtae Koo, Ki-Tae Ha, Seungtae Kim

**Affiliations:** Department of Korean Medical Science, School of Korean Medicine, Pusan National University, Yangsan, Republic of Korea; Georgia Regents University, UNITED STATES

## Abstract

Recent studies have shown that Korean Red Ginseng (KRG) suppresses dopaminergic neuronal death in the brain of a Parkinson’s disease (PD) mouse model, but the mechanism is still elusive. Using a 2-dimensional electrophoresis technique, we investigated whether KRG can restore the changes in protein expressions in the striatum (ST) of 1-methyl-4-phenyl-1,2,3,6-tetrahydropyridine (MPTP)-injected mice. Male C57BL/6 mice (9 weeks old) were injected with 20 mg/kg MPTP intraperitoneally four times at 2-h intervals. KRG (100 mg/kg) was orally administered once a day for 3 days from one hour after the first MPTP injection. Two hours after the third KRG administration a pole test was performed to evaluate motor function, after which the brains were immediately harvested. Survival of dopaminergic neurons in the nigrostriatal pathway and protein expression in the ST were measured by immunohistochemistry and 2-dimensional electrophoresis. KRG suppressed MPTP-induced behavioral dysfunction and neuronal death in the nigrostriatal pathway. Moreover, 30 proteins changed by MPTP and KRG in the ST were identified and shown to be related to glycolysis/gluconeogenesis and neurodegenerative diseases including Alzheimer’s disease and PD. KRG has neuroprotective effects against MPTP toxicity and alleviates protein expression profiles related to enhancing energy metabolism in the ST of MPTP-treated mice.

## Introduction

Parkinson’s disease (PD) is a well-known neurodegenerative disease characterized by selective dopaminergic cell death in the substantia nigra (SN) and striatum (ST) [1]. Degeneration of the nigrostriatal pathway causes striatal dopamine deficiency, which leads to symptoms of PD [2]. The motor symptoms of PD are mainly due to dopaminergic neuronal degeneration in the SN. And recent studies have shown that PD develops cognitive impairments, which is related to the neuronal death in the ST [3].

Various animal models are used to investigate potential treatment strategies and the pathogenesis of PD, and these are generally made by injecting neurotoxins that target the selective destruction of the catecholaminergic system [4]. Among these, 1-methyl-4-phenyl-1,2,3,6-tetrahydropyridine (MPTP) is a well-known neurotoxin that destructs dopaminergic neurons in the nigrostriatal pathway. Therefore, MPTP-treated mice are generally used as an animal model for investigating PD [5].

*Panax ginseng Meyer* is a valuable herb in Asian countries that is used as a crude substance to inhibit inflammation and enhance immunity and vitality. It has recently been suggested as a potential neuroprotective agent against PD [6] because several studies showed that it has neuroprotective [7], anti-oxidative [8] and anti-inflammatory effects [9]. Korean Red Ginseng (KRG) is steamed and dried *Panax ginseng Meyer* for lengthy preservation, and our previous study showed that the administration of KRG suppressed dopaminergic neuronal death in SN and ST against MPTP toxicity [7]. However the mechanism is still elusive because of a lack of research.

Two-dimensional electrophoresis is a widely used technique to analyze protein expressions in cells or tissues that is able to determine and analyze thousands of different proteins [10]. Using this technique, we investigated whether KRG administration can recover MPTP-induced proteomic alterations in the ST of mice.

## Materials and Methods

### Animals and groups

This study was approved by the Pusan National University Institutional Animal Care and Use Committee (PNU-2014-0538). Male nine-week-old C57BL/6 mice (Orientbio Inc., Seongnam, Korea) weighing 20–23 g were housed in a Plexiglas cage (200 mm X 320 mm X 145 mm, 3 mice in each cage) at room temperature (22 ± 2°C) under a standard 12-h light/dark cycle with unlimited access to a standard laboratory diet (Orientbio Inc.) and water. The animals were handled in accordance with the current guidelines established in the National Institutes of Health Guide for the Care and Use of Laboratory Animals (NIH Publication No. 85–23, 1985). The physical condition of the mice were monitored every other day in the adaptation period and every day in the experimental period. Humane endpoints for our study was as follows: 1) Weight loss more than 20% 2) No food intake more than 3 days 3) Diarrhea more than 3 days 4) Severe tremor or motor dysfunction not enough to evaluate their behaviors. None of mice died, became ill severely or reached a humane endpoint during the experiment. For immunostaining, mice were anesthetized with isoflurane then sacrificed by perfusion. For 2-dimensional electrophoresis and Western blotting, mice were sacrificed with CO_2_ gas.

Mice were randomly attached to three groups (n = 9 at each group): a saline-injected group (Saline), a MPTP-injected group (MPTP) and a MPTP-injected plus 100 mg/kg KRG-treated group (MPTP+KRG).

### MPTP injection and KRG administration

All mice except those in the saline group were injected with MPTP-HCl (20 mg/kg; Sigma, St. Louis, MO, USA) intraperitoneally four times at 2 h intervals (total 80 mg/kg) [11]. Mice in the saline group were injected with vehicle (normal saline) on the same schedule.

The KRG extract in this study was obtained from the Korea Ginseng Corporation (Daejeon, Korea). Briefly, ginseng was steamed at 90°C–100°C under no pressure for 3 h, dried at 50°C –80°C, and extracted three times with circulating hot water at 85°C –90°C for 8 h. The water content of the pooled extract was 36% of the total weight. Analysis of the content of crude ginsenoside in the extract by high-performance liquid chromatography revealed that it contained 6.92 mg/g Rb1, 2.68 mg/g Rb2, 3.24 mg/g Rc, 0.94 mg/g Rd, 1.40 mg/g Re, 1.03 mg/g Rf, 1.12 mg/g Rg1, 1.23 mg/g Rg2s, 1.03 mg/g Rg3r, 1.98 mg/g Rg3s, 0.89 mg/g Rh1 and other minor ginsenosides.

The KRG extract was diluted with sterilized mineral water to the appropriate concentrations. One hour after the first MPTP injections, mice in the MPTP+KRG group were orally administered the KRG extract (100 mg/kg) at 24 h intervals for 3 consecutive days. Mice in other groups were administered the same amount of vehicle on the same schedule [7].

### Pole test

Mice (n = 9 at each group) were mounted head-downwards near the top of a rough-surfaced wood pole (10 mm in diameter and 55 cm in height), and the time taken to reach the bottom of the pole was measured [12]. The test was repeated three times at 30 s time intervals, after which behavioral change was evaluated according to the average of the three times. The test was performed one day before MPTP injection (day 0) and 2 h after the last oral administration of KRG (day 3).

### Immunohistochemistry

After the last pole test, mice (n = 6 at each group) were perfused with 4% paraformaldehyde dissolved in 0.1 M phosphate buffer, and the brain was quickly harvested, post-fixed in the 4% paraformaldehyde buffer for 48 hours and immersed in 30% sucrose solution for storage at 4°C prior to sectioning. Frozen sections were cut to a thickness of 35 μm using a cryostat Leica CM3050S (Leica Microsystems, Wetzlar, Germany). The SN sections were located between AP –3.08 and –3.28 mm from the bregma and the ST sections were between AP +0.48 and +0.68 mm. The sections were incubated with 1% H_2_O_2_ in 0.05 M phosphate-buffered saline for 15 min, followed by 0.3% Triton X-100 and 3% normal blocking serum in PBS at room temperature for 1 h, then stained overnight at room temperature using an anti-tyrosine hydroxylase (TH, 1:500; Santa Cruz Biotechnology, Santa Cruz, CA, USA) primary antibody. The next day, sections were incubated with Vectastain Elite ABC reagents (Vector Laboratories Inc., Burlingame, CA) at room temperature for 1 h, then incubated with a diaminobenzidine substrate kit (Vector Laboratories Inc.) for 5 min. The tissues were subsequently mounted on gelatin-coated slides, air-dried, dehydrated, and coverslipped. Histological pictures were taken by an Axio Scope.A1 microscope (Zeiss, Germany) and an AxioCam ICc3 camera (ZEISS). The survival of dopaminergic neurons in the SN was evaluated by the number of TH-positive neuronal cells. An independent observer without knowing the expected results manually counted TH-positive neurons bilaterally in five continuous SN sections, and the cell counting was confirmed three times to validate the data. The survival of dopaminergic neurons in ST was evaluated by the mean value of optical density in ST using an Image-Pro Plus 6.0 (Media Cybernetics, Silver Spring, MD, USA).

### Two-dimensional protein electrophoresis

The tissues of ST were harvested instantly after the last pole test (n = 3 at each group) and kept at -80°C until use. Next, tissues were homogenized by a motor-driven homogenizer (PowerGen125, Fisher Scientific, Pittsburgh, PA, USA) in sample lysis solution composed with 7 M urea and 2 M thiourea containing 4% (w/v) 3-[(3-cholamidopropy)dimethyammonio]-1-propanesulfonate, 1% (w/v) dithiothreitol and 2% (v/v) pharmalyte and 1 mM benzamidine. After centrifugation at 15,000 × g for 1 h at 15°C, insoluble material was discarded and the soluble fraction was used for 2-dimensional electrophoresis.

Immobilized pH gradient dry strips (4–10 NL IPG, 24cm, Genomine, Korea) were equilibrated for 12 hours with 7M urea, 2M thiourea containing 2% 3-[(3-cholamidopropy)dimethyammonio]-1-propanesulfonate, 1% dithiothreitol, and 1% pharmalyte and loaded with 200 μg of sample. Isoelectric focusing was then performed at 20°C using a Multiphor II electrophoresis unit (Amersham Biosciences, Uppsala, Sweden) according to the manufacturer's protocols. Briefly, the voltage was linearly increased from 150 to 3,500 V over 3 h for sample entry, then maintained at 3,500 V at 96 kVh until focusing was complete. Prior to the second dimension, strips were incubated in equilibration buffer (50 mM Tris-Cl, pH 6.8 containing 6 M urea, 2% SDS and 30% glycerol) for 10 min, first with 1% DTT and then with 2.5% iodoacetamide. Equilibrated strips were inserted onto SDS-PAGE gels (20 x 24cm, 10–16%) and run at 20°C for 1,700 Vh using a Hoefer DALT 2D system (Amersham Biosciences), after which they were silver stained [13].

Using the PDQuest (version 7.0, BioRad, Hercules, CA, USA) software, quantitative analysis of digitized images was conducted according to the manufacturer’s instructions. The quantity of each spot was normalized by total valid spot intensity, and the spots that showed a change of more than 1.5-fold in expression compared with the control or normal sample were selected. Clustering analysis was then performed on a correlation matrix for both mice and proteins.

### Protein identification

For protein identification by peptide mass fingerprinting, protein spots were excised, digested with trypsin (Promega, Madison, WI, USA), mixed with α cyano-4-hydroxycinnamic acid in 50% acetonitrile/0.1% trifluoroacetic acid, and subjected to MALDI-TOF analysis (Microflex LRF 20, Bruker Daltonics, Billerica, MA, USA). Spectra were collected from 300 shots per spectrum over a m/z range of 600 to 3000 and calibrated by two point internal calibration using Trypsin auto-digestion peaks (m/z 842.5099, 2211.1046). A peak list was generated using Flex Analysis 3.0. The threshold used for peak-picking was as follows: 500 for minimum resolution of monoisotopic mass, 5 for S/N. The search program MASCOT (http://www.matrixscience.com/) was used for protein identification by peptide mass fingerprinting. The following parameters were used for the database search: trypsin as the cleaving enzyme, a maximum of one missed cleavage, iodoacetamide (Cys) as a complete modification, oxidation (Met) as a partial modification, monoisotopic masses, and a mass tolerance of ± 0.1 Da. peptide mass fingerprinting acceptance criteria was based on probability scoring. Pathway analysis of all metabolic and Kyoto Encyclopedia of Genes and Genomes (KEGG) pathways was performed using the pathway module.

### Western blotting

Equal amounts of protein (30 μg) from each sample were separated on 12% SDS-polyacrylamide gels and transferred to nitrocellulose membranes. The membrane was then blocked with 5% bovine serum albumin in tris-buffered saline containing 0.1% Tween-20 for 1 h at room temperature and incubated overnight at 4°C with anti-succinate dehydrogenase complex, subunit A (SDHA; Abcam, Cambridge, UK), or p44/42 mitogen-activated protein kinase (MAPK; Cell signaling Technology, Beverly, MA, USA) antibodies diluted 1:1000. The membrane was then incubated with horseradish peroxidase-conjugated secondary anti-rabbit antibody (1:1000, Santa Cruz Biotechnology) at room temperature for 1 h. Bands were detected using an enhanced chemiluminescence detection kit (Thermo Scientific, Rockford, IL, USA), after which these blots were re-probed with rabbit monoclonal anti-β-actin antibody (1:1000; Santa Cruz Biotechnology) as a loading control for all experiments. Quantification of immunoreactivity corresponding to the total bands was performed by densitometric analysis using an Image Quant LAS 4000 (Fujifilm, Tokyo, Japan).

### Statistical analysis

All data are expressed as the means ± standard deviation and analyzed by one-way analysis of variance with the Neuman-Keuls post hoc test. All statistical testing was performed using Prism 5 for Windows (GraphPad Software Inc., La Jolla, CA, USA) and statistical significance was set at *p* < 0.05.

## Results

### Effect of KRG on MPTP-induced behavioral change

**Fig 1:**
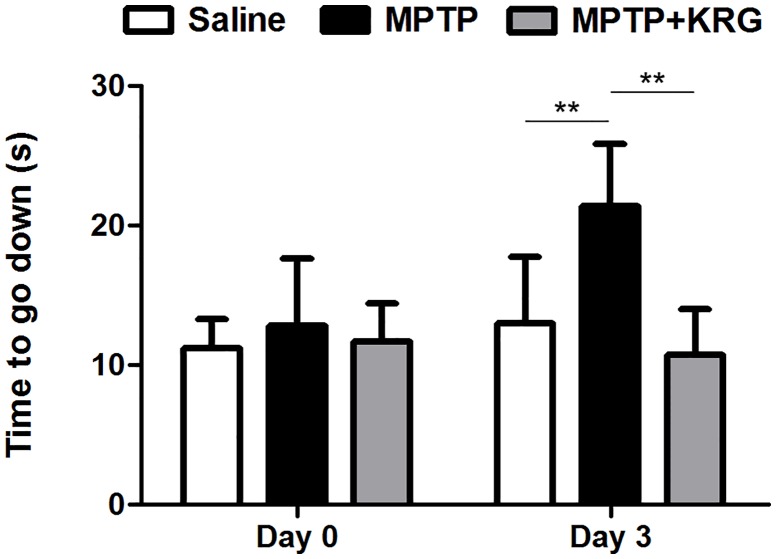
Results of the pole test. MPTP administration induced an increase in the descending time of mice, which was restored by oral administration of Korean Red Ginseng (KRG). Data are presented as the means ± the standard deviation. **p < 0.01. Saline, vehicle injected group; MPTP, MPTP injected group; MPTP+KRG, MPTP injected and KRG administrated group.

Before MPTP administration (day 0), descending times of mice in all groups were not significantly different. Three days after the MPTP administration (day 3), descending time in the MPTP group (21.40 ± 4.44 s) was significantly delayed relative to that in the saline group (13.02 ± 4.71 s, *p* < 0.01); however, that in MPTP+KRG group (10.72 ± 3.30 s) was significantly lower than that in the MPTP group (*p* < 0.01) and not significantly different from that in the saline group (Fig 1).

### Neuroprotective effect of KRG on MPTP-induced neuronal death in SN and ST

**Fig 2:**
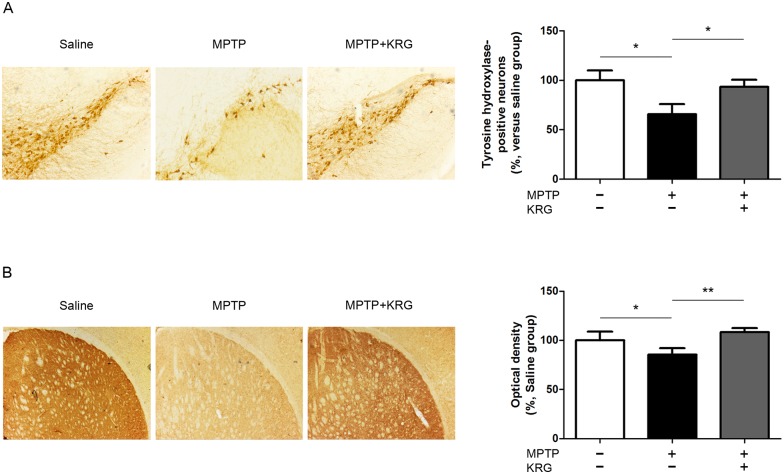
Expression of tyrosine hydroxylase in the nigrostriatal pathway on day 3. MPTP injection induced dopaminergic neuronal death in the substantia nigra (A) and the striatum (B), but KRG administration suppressed it. Scale bar, 200 μm. Data are presented as the means ± standard deviation. *p < 0.05 and **p < 0.01.

To evaluate the neuroprotective effect of KRG on MPTP-induced neuronal death, the number of TH-positive neurons in the SN was counted and the optical density of TH-positive fibers was evaluated. Compared to the number in the saline group (100.0 ± 9.90%), that in the MPTP group (72.15 ± 15.35%) was significantly reduced (*p* < 0.05). On the other hand, the number in the MPTP+KRG group (93.50 ± 7.04%) was significantly increased compared to that in the MPTP group (*p* < 0.05, Fig 2A).

TH immunoreactivity of the ST in the MPTP group (85.43 ± 6.71%) was significantly reduced relative to that in the saline group (100.0 ± 8.86%, *p* < 0.05). TH immunoreactivity in the MPTP+KRG group (108.2 ± 4.28%) was significantly increased relative to that in the MPTP group (*p* < 0.01), but not significantly different from that in the saline group (Fig 2B).

### Analysis and identification of differential protein expression

**Fig 3:**
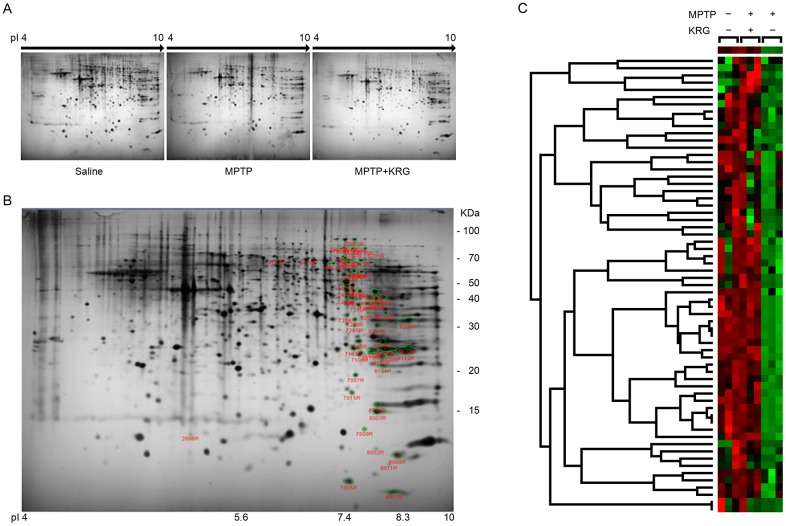
Protein profiles with two-dimensional electrophoresis. (A) Striatal tissue proteins obtained over different pI ranges. (B) Identified protein spots in gels. Differentially-expressed protein spots are circled with green. (C) Cluster matrix analysis. Red corresponds to high expression and green corresponds to low expression.

Over 690 spots were detected in the pH 4–10 interval upon silver staining (Fig 3A). Among these spots, a total of 63 were changed by more than 1.5 fold among the three experimental groups (Fig 3B). All protein spots were down-regulated by MPTP administration, but KRG administration restored the MPTP-induced protein down-regulation. Clustering analysis showed that the protein expressions in the MPTP+KRG group were more similar to those in the saline group than to those in the MPTP group (Fig 3C).

**Fig 4:**
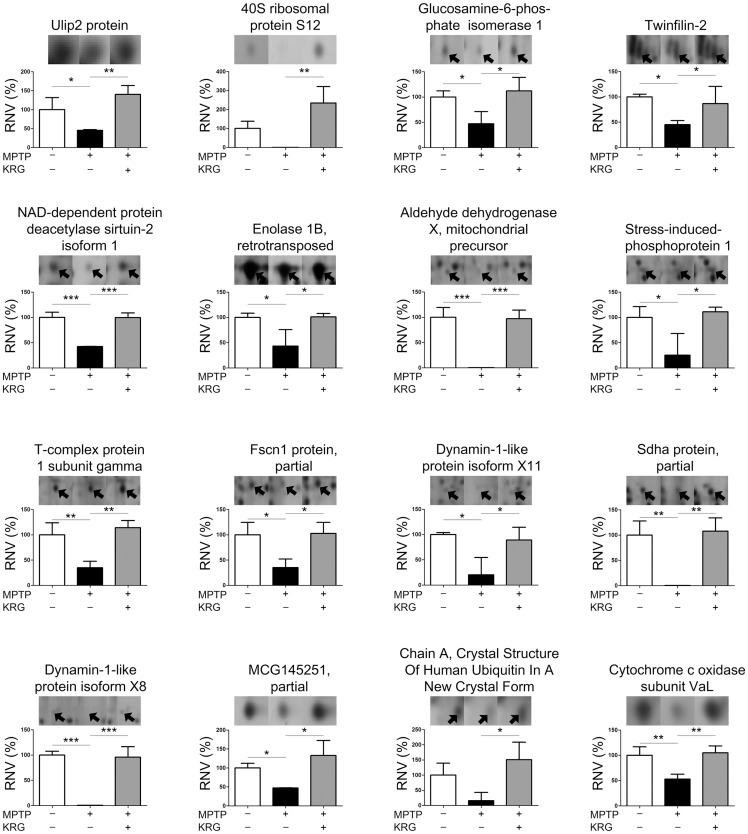
Protein levels differentially expressed in the striatum. The levels were illustrated as the relative normalized volumes (RNV) of the proteins. Bars represent the means ± standard deviation (n = 3 at each group). *p < 0.05, ** *p* < 0.01 and *** *p* < 0.001. Arrowheads represent protein spots.

**Fig 5:**
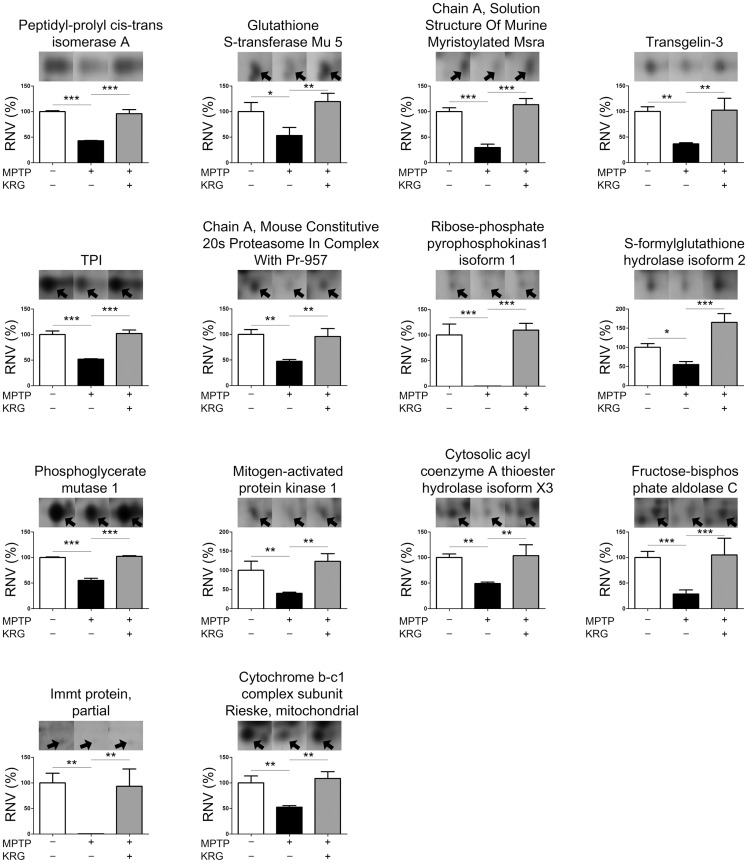
Protein levels differentially expressed in the striatum (continued).

Statistical analysis showed that 36 spots were significantly differentially expressed. MALDI-TOF analysis led to successful identification of 30 spots by interrogation of the NCBInr database (Table 1). The differential expression levels are illustrated in Figs 4 and 5. The spots were identified as ulip2 protein, 40S ribosomal protein S12, glucosamine-6-phosphate isomerase 1, twinfilin-2, NAD-dependent protein deacetylase sirtuin-2 isoform 1, retrotransposed enolase 1B, mitocholdrial precursor aldehyde dehydrogenase X, stress-induced-phosphoprotein 1, T-complex protein 1 subunit gamma, partial fscn1 protein, dynamin-1-like protein isoform X11, partial SDHA protein, dynamin-1-like protein isoform X8, partial MCG145251, crystal structure of human ubiquitin in a new crystal form, cytochrome c oxidase subunit VIaL, peptidyl-prolyl cis-trans isomerase A, glutathione S-transferase mu 5, solution structure of murine myristoylated msra, transgelin-3, TPI, mouse constitutive 20s proteasome in complex with Pr-957, ribose-phosphate pyrophosphokinase 1 isoform 1, s-formylglutathione hydrolase isoform 2, phosphoglycerate mutase 1, mitogen-activated protein kinase 1, cytosolic acyl coenzyme A thioester hydrolase isoform X3, fructose-bisphosphate aldolase C, partial immt protein and mitochondrial cytochrome b-c1 complex subunit rieske. The expression of all proteins was suppressed by MPTP, but KRG oral administration alleviated them.

**Table 1:** List of differentially expressed protein spots following MPTP and KRG administration.

Spot number	Protein name	Protein ID	Theoretical		Gel		Score	Sequence coverage (%)	No. of matched peptides	Fold change^a^	
			Mr (KDa)	pI	Mr (kDa)	pI				Saline/MPTP	MPTP+KRG/MPTP
5718	Ulip2 protein	CAA71370	62.5	6.0	71.3	6.8	282	59	26	-2.19	2.63
7009	40S ribosomal protein S12	NP_001007	14.9	6.8	13.4	7.7	68	45	6	-189.21	401.37
7205	Glucosamine-6-phosphate isomerase 1	NP_036067	32.7	6.1	30.9	7.6	130	45	12	-2.12	2.40
7307	Twinfilin-2	BAC39867	39.7	6.3	40.6	7.6	88	40	8	-2.22	1.91
7312	NAD-dependent protein deacetylase sirtuin-2 isoform 1	NP_071877	43.8	5.2	38.1	7.8	328	63	22	-2.36	2.26
7404	Enolase 1B, retrotransposed	NP_001020559	47.5	6.4	52.2	7.5	217	49	17	-2.31	2.32
7506	Aldehyde dehydrogenase X, mitochondrial precursor	NP_082546	58.1	6.6	58.4	7.6	181	38	17	-346.15	327.72
7605	Stress-induced-phosphoprotein 1	NP_058017	63.2	6.4	69.8	7.5	185	39	22	-3.98	4.13
7606	T-complex protein 1 subunit gamma	NP_033966	61.2	6.3	67.7	7.5	206	42	23	-2.86	3.11
7609	Fscn1 protein, partial	AAH37137	52.2	6.6	60.3	7.6	407	62	25	-2.83	2.76
7701	Dynamin-1-like protein isoform X11	XP_006522701	65.9	7.2	81.4	7.4	143	25	13	-4.91	4.35
7702	SDHA protein, partial	AAH11301	73.4	7.1	73.0	7.4	273	50	25	-221.93	229.26
7808	Dynamin-1-like protein isoform X8	XP_006522698	80.9	6.3	83.9	7.7	124	21	11	-148.51	140.53
8003	MCG145251, partial	EDL15910	17.4	6.8	15.3	8.0	187	66	12	-2.11	2.69
8007	Chain A, crystal structure of human ubiquitin in a new crystal form	3ONS_A	8.2	5.7	8.5	8.2	125	81	6	-6.37	7.52
8008	Cytochrome c oxidase subunit VIaL	AAA17836	9.7	6.6	11.1	8.3	94	93	5	-1.89	1.93
8018	Peptidyl-prolyl cis-trans isomerase A	NP_032933	18.1	7.7	16.0	7.9	171	74	13	-2.34	2.23
8102	Glutathione S-transferase mu 5	NP_034490	27.0	6.8	24.9	7.9	202	70	21	-1.88	2.08
8103	Chain A, solution structure of murine myristoylated msra	2L90_A	23.9	6.8	23.5	8.0	71	46	7	-3.37	3.73
8104	Transgelin-3	NP_062728	22.6	6.8	21.6	8.0	167	69	16	-2.74	2.76
8106	TPI	AAC36016	27.0	6.9	25.1	8.1	219	61	16	-1.93	1.94
8108	Chain A, mouse constitutive 20s proteasome in complex with Pr-957	3UNB_A	26.0	8.4	23.9	8.1	128	46	10	-2.11	2.06
8201	Ribose-phosphate pyrophosphokinase 1 isoform 1	NP_002755	35.3	6.5	34.8	7.8	193	48	15	-422.54	421.09
8204	S-formylglutathione hydrolase isoform 2	NP_058599	31.9	6.7	31.0	7.9	109	45	9	-1.82	3.00
8212	Phosphoglycerate mutase 1	NP_075907	28.9	6.7	26.8	8.0	253	74	17	-1.81	1.85
8301	Mitogen-activated protein kinase 1	NP_036079	41.6	6.5	40.6	7.8	159	40	16	-2.49	2.77
8302	Cytosolic acyl coenzyme A thioester hydrolase isoform X3	XP_006539231	36.4	7.2	40.2	7.8	120	36	13	-2.04	2.13
8304	Fructose-bisphosphate aldolase C	NP_033787	39.8	6.7	40.9	8.0	154	55	14	-1.57	1.56
8701	Immt protein, partial	AAI08357	50.2	5.7	77.5	7.9	112	31	10	-201.79	193.21
9112	Cytochrome b-c1 complex subunit rieske, mitochondrial	NP_079986	29.6	839	24.6	8.5	90	25	8	-1.91	2.12

^a^. Calculated by dividing the higher by the lower number in each pair of NV values, and preceded by a minus (-) symbol when the lower was that of the MPTP-treated sample.

### KEGG pathway analysis

To investigate functions of the proteins, KEGG pathway analysis was performed. Eleven proteins of the 30 proteins were mapped onto the KEGG database. The glycolysis/gluconeogenesis pathway was the most statistically significant, followed by Alzheimer’s disease, oxidative phosphorylation and Parkinson’s disease pathways (Table 2).

**Table 2:** KEGG pathway list of 12 proteins which were down-regulated in MPTP versus saline and up-regulated in MPTP+KRG versus MPTP.

KEGG pathway	Protein ID	Number of molecules		*P* value
		Mapping	All	
Glycolysis / Gluconeogenesis	NP_075907; NP_033787; AAC36016; NP_082546; NP_001020559	5	68	0.00003
Alzheimer's disease	NP_036079; NP_079986; AAH11301; AAA17836	4	182	0.01296
Oxidative phosphorylation	NP_079986; AAH11301; AAA17836	3	130	0.04968
Parkinson's disease	NP_079986; AAH11301; AAA17836	3	133	0.05176
Huntington's disease	NP_079986; AAH11301; AAA17836	3	183	0.09062
Prion diseases	NP_036079; NP_058017	2	35	0.09337
Fructose and mannose metabolism	NP_033787; AAC36016	2	57	0.09845

### Verification of protein changes by Western blotting

**Fig 6:**
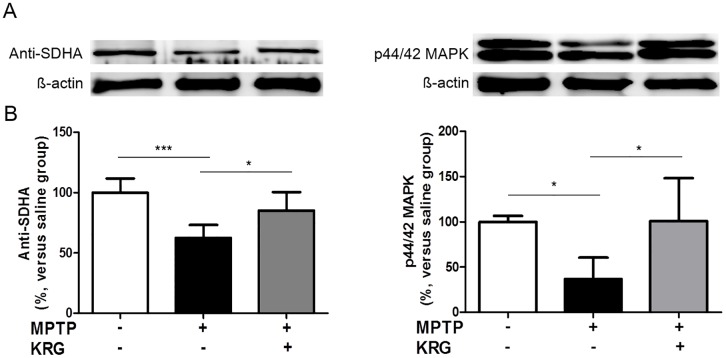
Confirmation of the proteomic analysis using Western blotting. The expression of p44/42 MAPK and SDHA showed the same tendencies as the results of 2-dimensional electrophoresis analysis. All data are shown as the means ± standard deviation (n = 3 in each group). *p < 0.05 and ***p < 0.001.

MAPK1 and SDHA were selected as representative proteins to confirm the reliability of the proteomic analysis, and their expressions were confirmed by Western blotting. The results of the Western blots were similar to those of 2-dimensional electrophoresis (Fig 6).

## Discussion

This study demonstrated that KRG extract suppressed MPTP-induced dopaminergic neuronal death in the nigrostriatal pathway and restored the MPTP-induced proteomic changes in the ST. KEGG pathway analysis showed that the changed proteins play a major role in the pathways of glycolysis/gluconeogenesis, oxidative phosphorylation and neurodegenerative diseases.

Ginseng has traditionally been used to tonify the healthy qi, which means that KRG may help enhance physical and mental activities. Actually, several studies showed that ginseng alleviates motor dysfunction of rats that have undergone spinal cord injury [14] and KRG improves motor and cognitive functions in normal subjects [15], which can be considered a basis of its tonifying effects.

To improve various functions in the body, it is essential to up-regulate energy metabolism because our body spends energy when we perform physical and mental activities. Therefore, KRG should be able to regulate energy metabolism if it can improve body functions. Glycolysis and gluconeogenesis are included in the metabolism of common monosaccharides, which play a major role in the regulation of energy metabolism. Oxidative phosphorylation is also an important metabolic pathway to release energy. In this study, MPTP administration suppressed the motor function and expression of five proteins related to glycolysis and gluconeogenesis and three proteins related to oxidative phosphorylation, but their suppression was alleviated by KRG oral administration. These findings suggest that KRG administration can up-regulate body functions by enhancing the action of energy metabolism.

KEGG pathway analysis revealed that KRG also influences neurodegenerative disease-related pathways by alleviating the expression of four proteins suppressed by MPTP administration, MAPK1, SDHA, mitocholdrial cytochrome b-c1 complex subunit Rieske and cytochrome c oxidase (COX) subunit VIaL. Recent studies have shown that succinate dehydrogenase and SDHA were suppressed in the brains of neurodegenerative disease patients or animal models. For example, the activity of succinate dehydrogenase was decreased in ST of a Huntington’s disease rat model [16], and the expression of SDHA was reduced in dopaminergic substantia nigra neurons from PD patients [17]. Cytochrome b-c1 complex subunit Rieske is a subunit of complex III of the mitrochondrial respiratory chain, which plays an important role in synthesizing ATP [18]. COX is complex IV of the mitochondrial respiratory chain, which is composed of 14 protein subunits. Several studies have shown that COX was decreased in brains subject to neurodegenerative diseases. For example, the activity of COX was reduced in the brain of Alzheimer’s disease patients [19], and MPTP administration suppressed the expression of COX subunit 5b in ST of C57/BL6 mice [12]. In this study, MPTP administration suppressed the expression of SDHA, cytochrome b-c1 complex subunit Rieske and COX subunit VIaL, which are constituents of the mitochondrial respiratory chain. However, their suppression was alleviated by KRG administration, indicating that KRG administration protects the complexes of the mitochondrial respiratory chain against MPTP toxicity.

MAPK1 is involved in various cellular processes including proliferation, differentiation, transcription and development [20], and fructose-bisphosphate aldolase C is expressed in Purkinje cells of the brain, which provide neuroprotection after trauma and excitotoxicity [21]. In this study, MPTP down-regulated the expression of MAPK1 and fructose-bisphosphate aldolase C, indicating that it suppresses neuroprotection-related factors in dopaminergic neurons in ST. The MPTP-induced downregulation was alleviated with KRG administration, suggesting that KRG protects dopaminergic neurons in the ST by increasing neuroprotection-related factors such as MAPK1 and fructose-bisphosphate aldolase C against MPTP toxicity.

Although this study showed that KRG enhanced the MPTP-induced down-regulation of proteins related to energy metabolism and neurodegenerative diseases, it should be noted that it had several limitations. First, the study showed proteomic changes in response to MPTP and KRG in the early stage, so the reasons for the changes in the late stage are still elusive. Second, positive control was not used to investigate the neuroprotective effect of KRG. Third, there is a possibility that KRG interfere with the generation of the active 1-methyl-4-phenylpyridinium toxin from MPTP. Despite these limitations, KRG has the potential for treatment of PD.

## Conclusion

KRG suppressed MPTP-induced movement impairment and dopaminergic cell death in the nigrostriatal pathway and restored the expression of proteins down-regulated by MPTP in the ST that were related to energy metabolism, mitochondrial respiratory chain and neuroprotection. There results indicate that KRG protects dopaminergic neurons in the ST by ameliorating MPTP-induced impairments in the animal model for PD.
